# Primary pulmonary hyalinizing clear cell carcinoma with *EWSR1* gene translocation: a case report

**DOI:** 10.3389/fonc.2024.1509132

**Published:** 2024-12-11

**Authors:** Can-hui Jian, Shuai Luo, Jin-jing Wang

**Affiliations:** Pathology Department, Affiliated Hospital of Zunyi Medical University, Zunyi, Guizhou, China

**Keywords:** pulmonary tumor, hyalinization, clear cell carcinoma, diagnostic, clinicopathological features

## Abstract

**Background:**

Primary pulmonary hyalinizing clear cell carcinoma (HCCC) is a rare type of primary salivary gland-type tumor of the lung. HCCC is characterized by unique pathological features, including nests, cords, or trabeculae of clear or eosinophilic tumor cells infiltrating a mucinous or hyalinized stroma. Additional analyses of this carcinoma have revealed positive epithelial markers via immunophenotyping and *EWSR1* gene translocation through genetic testing. However, the morphology of HCCC has been found to change during bronchoscopic biopsy, suggesting certain challenges for its clinical diagnosis and treatment.

**Case presentation:**

A 47-year-old female patient presented with a 2-month history of cough, sputum production, and dyspnea. A chest CT scan found a nodular soft tissue density shadow in the lower segment of the trachea. Subsequently, the patient underwent tumor resection via combined flexible and rigid bronchoscopy. Postoperative pathological examination, including immunohistochemistry and molecular testing, confirmed an *EWSR1* gene translocation. The final pathological diagnosis was primary pulmonary HCCC. A follow-up at 6 months post-surgery showed mediastinal lymph node metastasis.

**Conclusions:**

Primary pulmonary HCCC is an extremely rare, low-grade malignant epithelial tumor of the lung, which has a notably difficult clinical diagnosis and treatment due to the absence of a standard treatment protocol. This case report presents a patient with primary pulmonary HCCC confirmed by molecular testing, aiming to raise awareness about this tumor among physicians and provide valuable clinical references.

## Background

Hyalinizing clear cell carcinoma (HCCC) typically occurs in the salivary glands, while its primary pulmonary occurrence is extremely rare, with a rate of 0.1% among primary pulmonary tumors. tumors (1). Primary pulmonary HCCC was first reported in 2015 and was newly proposed as a rare subtype of lung cancer in the 5th edition of the WHO Classification of Thoracic Tumors (2).

This tumor exhibits indolent growth and a slow clinical course, with characteristic infiltration by clear or eosinophilic tumor cells within a markedly hyalinized stroma and an EWSR1 gene rearrangement (1). Previous reports have confirmed that the histological and immunogenetic features of primary pulmonary HCCC are similar to those of primary salivary gland HCCC (2). Here, we present a case of a patient with primary pulmonary HCCC.

## Case presentation

**Figure 1:**
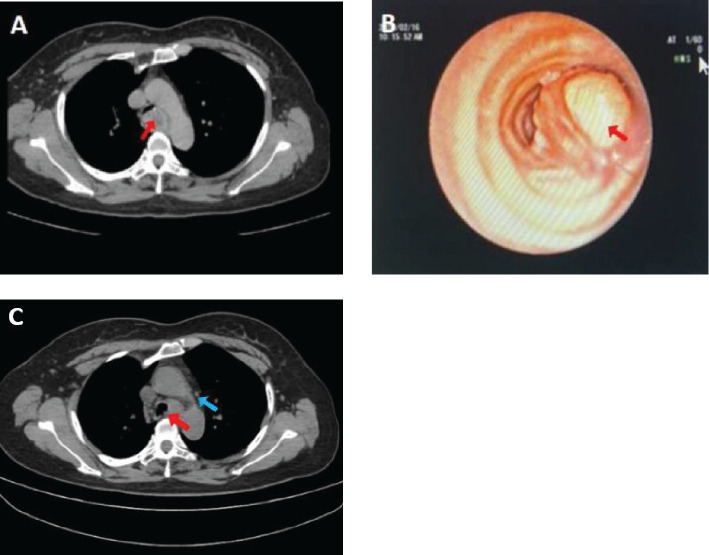
Chest CT scan shows a nodular soft tissue density shadow on the left wall of the lower segment of the trachea, with a maximum diameter of 2.8 cm [**(A)**, arrow]. The bronchoscopic view reveals a mass obstructing the lumen of the anterior basal segment of the lower lobe of the left lung [**(B)**, arrow]. Reexamination with PET-CT scan demonstrates a nodular shadow in the lower trachea, with a maximum diameter of 1.3 cm [**(C)**, red arrow] and multiple enlarged mediastinal lymph nodes (Panel **C**, blue arrow).

The patient was a 47-year-old Chinese woman who presented with cough, sputum production, and inspiratory dyspnea following a cold 2 months ago. She had no history of lung cancer, tuberculosis, or other significant pulmonary diseases. Additionally, no family history of other malignant tumors, long-term farming, and carcinogenic risk factors were noted. Chest CT imaging revealed a nodular soft tissue density shadow of approximately 28 mm × 23 mm in size on the left wall of the lower trachea (**Figure 1A**). The lesion showed clear boundaries and a relatively uniform density on plain CT scan with a value of approximately 27 HU. Tests for lung cancer markers found a slightly elevated level of carbohydrate antigen 125 at 70.26 U/ml. Subsequently, the patient underwent bronchoscopy, hard tracheoscopic mass resection and stenting under general anesthesia, and it was impossible to determine whether the resection margin was negative. Bronchoscopy further revealed that the tumor had completely obstructed the lumen of the middle and lower trachea (**Figure 1B**), with a smooth surface and easy bleeding upon contact. The patient completed six cycles of postoperative chemotherapy (albumin-bound paclitaxel combined with cisplatin). A follow-up at 6 months post-surgery showed mediastinal lymph node metastasis (**Figure 1C**).

**Figure 2:**
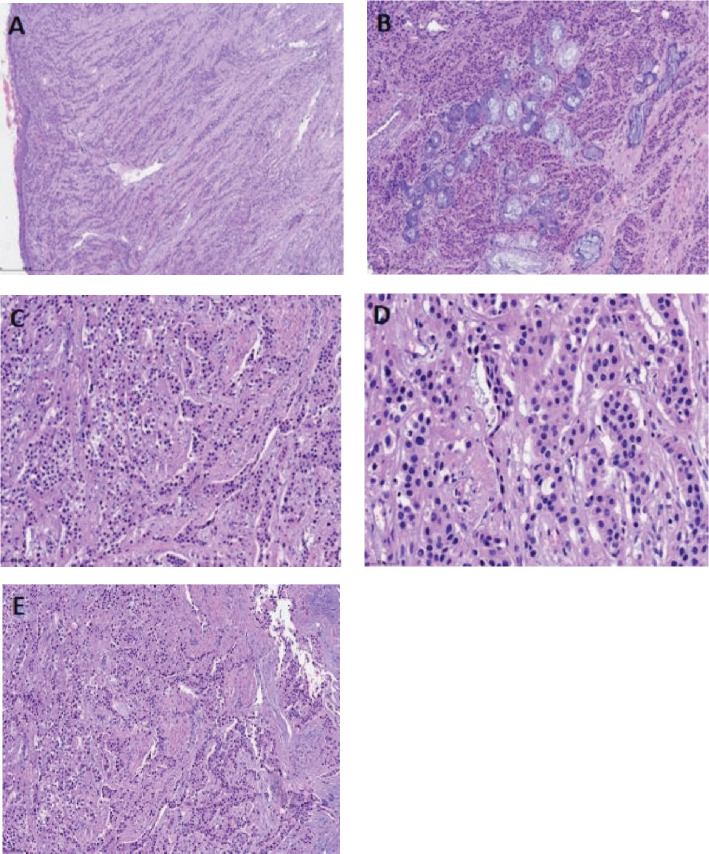
Hematoxylin and eosin (HE) staining displays a submucosal tumor in the respiratory tract [**(A)**, scale bar: 500 µm]. Some tumor cells involve the submucosal glands, with the tumor cells arranged in cords, nests, trabeculae, or solid patterns [**(B)**, scale bar: 200 µm]. The tumor cells exhibit a mild morphology, round or oval shape, and uniform size but no significant atypia. Some tumor cells have eosinophilic cytoplasm, while others have clear cytoplasm [**(C)**, scale bar: 100 µm]. The nuclei are small with fine chromatin [**(D)**, scale bar: 50 µm] and occasional mitotic figures, but no prominent nucleoli or necrosis are observed. Focal squamous metaplasia is present. The tumor stroma exhibits sclerosis with hyalinization and myxoid changes [**(E)**, scale bar: 100 µm].

Gross examination of the bronchoscopic resection specimen demonstrated a mass of gray-white fragmented tissue, approximately 3 cm × 2.5 cm × 0.5 cm in size and a medium consistency. Histopathological examination under low magnification (**Figure 2A**) indicated that tumor cells in the submucosa were arranged in cords, nests, trabeculae, or solid patterns (**Figure 2B**), resembling glandular structures. Additional histopathological assessment under high magnification showed that the tumor cells had a mild morphology, exhibiting a round or oval shape with consistent size and no prominent atypia. Moreover, some tumor cells possessed eosinophilic cytoplasm and others had clear cytoplasm (**Figure 2C**), along with fine chromatin and occasional mitotic figures (**Figure 2D**). Furthermore, no prominent nucleoli or necrosis was observed, while focal areas of squamous differentiation were noted. Lastly, the tumor stroma showed sclerosis with hyalinization (**Figure 2E**), and perineural invasion was detected.

**Figure 3:**
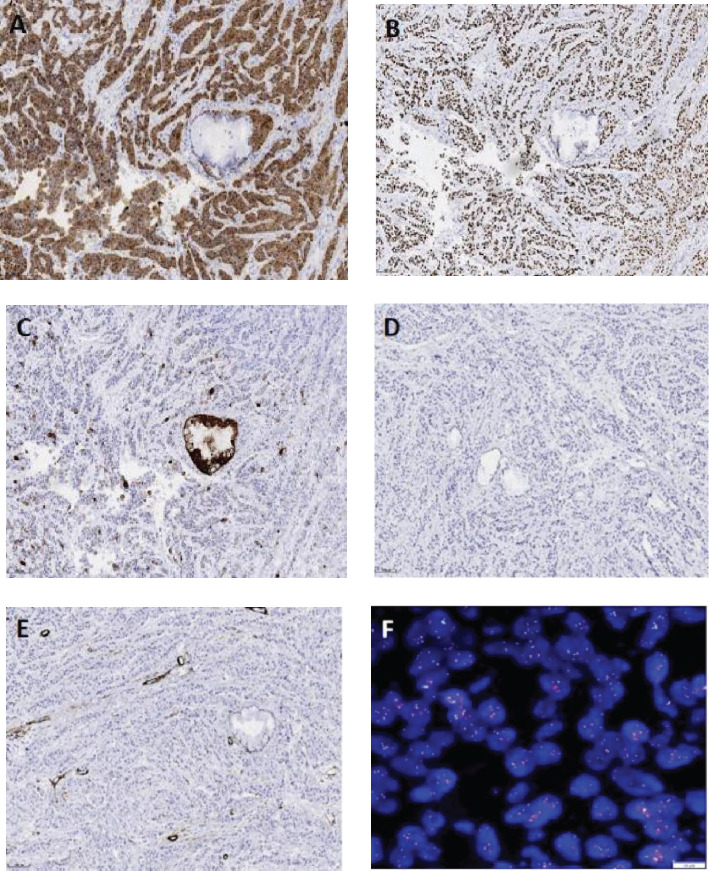
Immunohistochemistry (EnVision method, scale bar: 100 µm) of the tumor cells shows a diffuse expression of CK5/6 **(A)** and P63 **(B)**, focal expression of CK7 **(C)**, and no expression of TTF-1 **(D)**, SMA **(E)**, CgA, or CD56. Fluorescence *in situ* hybridization reveals EWSR1 gene translocation [**(F)**, scale bar: 10 µm].

Immunohistochemical staining showed positivity for CK5/6 (**Figure 3A**), p63 (**Figure 3B**), and CK, with a focal positivity for CK7 (**Figure 3C**). Conversely, negative staining was noted for TTF-1 (**Figure 3D**), Napsin A, Syn, CgA, CD56, CD117, SMA (**Figure 3E**), and S-100. The Ki-67 proliferation index was approximately 5%.

According to the histomorphological and immunophenotyping results, mucoepidermoid carcinoma or squamous cell carcinoma (SCC) could not be excluded in patient. The diagnosis was confirmed by performing fluorescence *in situ* hybridization (FISH). FISH demonstrated EWSR1 gene rearrangement (**Figure 3F**), with 88% of the cells showing split red and green signals. Although an EWSR1 gene rearrangement was identified in our patient, she refused to undergo sequencing or qRT-PCR testing. Therefore, the fusion partner of the EWSR1 gene was not determined. Nevertheless, a diagnosis of primary pulmonary HCCC was established based on the above results.

Diagnosis: Primary Pulmonary HCCC, stage T4N2M0 IIIB.

## Discussion and conclusions

HCCC was initially described as a salivary gland tumor in the oral and maxillofacial regions. However, HCCC has been recently found in other sites, including the lung. Nonetheless, primary salivary gland-type tumors of the lung are rare, with adenoid cystic and mucoepidermoid carcinomas being more common. In addition to the previously recognized pleomorphic adenoma, the 5th edition of the WHO Classification of Thoracic Tumors includes myoepithelioma, adenoid cystic carcinoma, mucoepidermoid carcinoma, and epithelial-myoepithelial carcinoma, as newly recognized and rare salivary gland-type tumors. Primary pulmonary HCCC, with ICD-O code 8310/3, is a low-grade malignant epithelial tumor associated with the small salivary glands in the submucosa of the trachea and bronchi (3). This tumor predominantly presents as a central endobronchial lesion, leading to common clinical symptoms of airway obstruction and even hemoptysis in severe cases. It is slightly more prevalent in females and is not associated with smoking history. Additionally, it exhibits indolent growth, slow progression, extremely low aggressiveness, and a generally favorable prognosis. Currently, primary pulmonary HCCC has no specific treatment protocol, with surgical resection as the primary clinical approach. Fewer than 30 cases of primary pulmonary HCCC have been reported worldwide to date (4), of which most were case reports. Among the reported patients, only one had peribronchial metastasis 16 years post-surgery. Furthermore, only one patient was definitively diagnosed with pulmonary HCCC before surgery (4), mostly as case reports. Among these, only one case reported peribronchial metastasis 16 years post-surgery. Only one out of the pulmonary HCCC reported in the literature was clearly diagnosed before surgery (4), and the rest of was clearly diagnosed after surgical resection, which indicated that the diagnosis of this tumor was challenging, and the preoperative diagnosis increased the difficulty.

Primary pulmonary HCCC exhibits mild, low-grade tumor cell morphology. This tumor is characterized by nests, trabeculae, and cords of tumor cells within mucinous or sclerotic hyalinized stroma. Moreover, the tumor cells demonstrate no pronounced atypia and have a uniform size with a round or oval shape. Most tumor cells have eosinophilic cytoplasm, while some may have pale, clear cytoplasm. These cells show no necrosis, and their nuclei have a relatively consistent size, with fine chromatin, occasional nucleoli, and rare mitotic figures.

Immunohistochemistry is valuable for diagnosing pulmonary HCCC, Immunohistochemistry is valuable for diagnosing pulmonary HCCC, which has immunophenotypic characteristics similar to those of salivary gland HCCC. The pulmonary HCCC cells express epithelial markers such as CK, EMA, CK5/6, P63, and P40 but do not express myoepithelial markers (including SMA, Calponin, and S-100), adenocarcinoma markers (e.g., TTF-1 and Napsin A), or neuroendocrine markers (such as CD56, CgA, and Syn). Molecular genetic examinations have revealed characteristic EWSR1 gene translocations, with most cases demonstrating EWSR1-ATF1 gene fusion (5, 6), whereas a few exhibit EWSR1-CREM (7), and IRF2-NTRK3 gene fusions (4), similar to head and neck HCCC. Presently, no studies have reported a difference in the prognosis between tumors with EWSR1-ATF1 and EWSR1-CREM gene fusions. However, EWSR1 gene translocations are also observed in clear cell sarcoma, desmoplastic small round cell tumor, Ewing sarcoma, and myxoid liposarcoma. In particular, EWSR1-ATF1 gene fusion has been detected in clear cell sarcoma, angiomatoid fibrous histiocytoma, and angiosarcoma, suggesting that this gene fusion is not unique to HCCC. Although HCCC is difficult to diagnose, second-generation sequencing, qRT-PCR, RNA-seq, and other methods can aid the diagnostic process and guide clinical medication (3).

According to the previous literature, specimens from bronchoscopic biopsy and surgical resection of primary pulmonary HCCC are often misdiagnosed as SCC, adenocarcinoma, mucoepidermoid carcinoma, or other carcinomas (8), underlining its high misdiagnosis rate. Therefore, differentiating HCCC from other primary and metastatic tumors is essential, and the following differential diagnoses can be considered:

1. Pulmonary SCC: SCC with clear cytoplasm can be challenging to differentiate from HCCC. SCC usually displays substantial cellular atypia, keratinization, squamous pearl formation, strong positivity for CK5/6, P63, and P40, weak positivity for CK7, negative staining for TTF-1 and Napsin A, and a high Ki-67 proliferation index. Additionally, SCC does not exhibit EWSR1 gene rearrangement.
2. Pulmonary adenocarcinoma or neuroendocrine tumors: Solid adenocarcinoma is positive for TTF-1 and Napsin A on immunohistochemistry, while neuroendocrine tumors are positive for CD56, CgA, and Syn. All these markers can be utilized to differentiate these two cancers.
3. Mucoepidermoid carcinoma: This tumor comprises mucin-secreting cells, epidermoid cells, and intermediate cells, which typically proliferate in solid, nested, glandular, or cystic patterns. This disease often shows stromal fibrosis and sclerosis as well as MAML2 gene rearrangement, which can be used in its differentiation (9).
4. Clear cell myoepithelial carcinoma: This tumor usually presents with pushing borders, grows in solid, trabecular, nested, or multinodular patterns, and potentially exhibits central necrosis. The tumor cells resemble clear cells and are surrounded by scant fibrous septa. Immunohistochemistry demonstrates positive expression of myoepithelial markers, aiding in the differential diagnosis of this tumor.
5. Clear cell acinic cell carcinoma: This tumor consists of acini or ducts, which are arranged in solid or microcystic patterns with empty lumen structures. The tumor cells have clear cytoplasm with filamentous content but without notable atypia, mitotic figures, or necrosis. Immunohistochemistry shows positive expression of DOG1 and SOX10, contributing to the differentiation of this carcinoma.
6. Metastatic clear cell carcinoma to the lung: Metastatic clear cell carcinoma from the kidney or ovary may lack hyalinized sclerotic stroma. Moreover, immunohistochemistry reveals positive expression of PAX8. Clinical history and imaging studies can further assist in differentiating this carcinoma.

Currently, no standard treatment exists for HCCC, with complete surgical resection being the main treatment strategy (10). According to the reports in earlier literature, most patients underwent complete surgical resection, and only one had laser excision under bronchoscopy (11). Most patients had a good prognosis, while a few experienced metastases (12).

In the absence of a specific standard treatment regimen, patients with rare lung cancer are mainly treated according to the treatment regimen for non-small cell lung cancer (13), However, the heterogeneity between various tumors can lead to the poor clinical efficacy of this treatment approach. The standard treatment for patients with advanced cancer involves platinum-based combination chemotherapy (14). In clinical practice, targeted therapies that may benefit from detecting matched biomarkers are more feasible. For example, current non-small cell lung cancer targets approved by the FDA include EGFR, MET14 exon-skipping mutation, KRAS mutation, VEGF mutation, BRAF V600E point mutation, ALK fusion, ROS 1 gene fusion, NTRK gene fusion, RET rearrangement, and immune checkpoint inhibitors, with the immune checkpoint inhibitors effectively stimulating anti-tumor immunity (15). Prior research has reported that the medication indication of a TRK inhibitor for patients with an IRF1-NTRK3 gene fusion can be confirmed by molecular testing (4). The development of sequencing technology has increased the exploration of targeted therapy for rare lung cancers, ultimately leading to improved treatment efficacy. In our case report, the patient underwent six cycles of combination chemotherapy according to the first-line chemotherapy regimen. However, reexamination with PET-CT revealed multiple nodular soft tissue shadows in the tracheal lumen and potential mediastinal lymph node metastasis, indicating that the tumor had high aggressiveness, recurrence and metastasis had developed over a short period, and the combined chemotherapy had a poor efficacy. According to the NCCN guidelines, we can further perform sequencing to determine whether the patient is suitable for neoadjuvant systemic treatment (immune checkpoint inhibitor combined with chemotherapy), optimize the chemotherapy plan based on the genetic testing results, and perform concurrent radiotherapy. After adjuvant treatment, we can evaluate the decline after induction therapy. feasibility of early stage surgery. According to the NCCN guidelines, we can further perform sequencing to determine whether the patient is suitable for neoadjuvant systemic treatment (immune checkpoint inhibitor combined with chemotherapy), optimize the chemotherapy plan based on the genetic testing results, and perform concurrent radiotherapy. After adjuvant treatment, we can evaluate the tumor after adjuvant treatment, and to determine the feasibility of surgery.

Although previous literature has reported a good prognosis of pulmonary HCCC, our patient experienced recurrence and metastasis in a short time after the operation. Thus, the tumor in our patient was more aggressive and possibly carried driver gene mutations. However, the patient underwent tumor resection only under bronchoscopy, which could not guarantee a negative margin. Additionally, no lymph node dissection was performed. Moreover, a positive surgical margin is associated with high-grade transformation (tumor necrosis, prominent nuclear atypia, high nuclear division on imaging, and a high proliferation index), as well as a heightened risk of tumor recurrence and metastasis (16).

HCCC typically occurs in the salivary glands, while primary HCCC in the lung shares similar histological, immunophenotypic, and molecular pathological characteristics with HCCC of the head and neck (17). However, the rarity of primary pulmonary HCCC increases the risk of its misdiagnosis or delayed diagnosis. Although primary HCCC has characteristic pathological features, its morphology can change during bronchoscopic biopsy. Furthermore, HCCC has no specific immunohistochemical markers, making it more likely to be misdiagnosed with tumor cells having squamous and mucinous differentiation. Immunohistochemistry and molecular testing can aid in the diagnosis and treatment of HCCC. Tumors with high-grade nuclear features, necrosis, high proliferation index, and numerous pathological mitotic figures require careful monitoring and close follow-up due to the potential for recurrence and metastasis. Currently, extremely few cases of pulmonary HCCC have been reported. Thus, more cases are needed for in-depth research on its pathogenesis, treatment efficacy, and prognosis evaluation.

## Data Availability

The original contributions presented in the study are included in the article/supplementary material. Further inquiries can be directed to the corresponding author.
